# High‐throughput compound screen reveals mTOR inhibitors as potential therapeutics to reduce (auto)antibody production by human plasma cells

**DOI:** 10.1002/eji.201948241

**Published:** 2019-11-14

**Authors:** Paul Tuijnenburg, Daan J. aan de Kerk, Machiel H. Jansen, Ben Morris, Cor Lieftink, Roderick L. Beijersbergen, Ester M.M. van Leeuwen, Taco W. Kuijpers

**Affiliations:** ^1^ Emma Children's Hospital Amsterdam UMC, University of Amsterdam Department of Pediatric Immunology Rheumatology and Infectious diseases Amsterdam The Netherlands; ^2^ Amsterdam UMC, University of Amsterdam Department of Experimental Immunology Amsterdam Infection & Immunity Institute Amsterdam The Netherlands; ^3^ Division of Molecular Carcinogenesis and NKI Robotics and Screening Center Netherlands Cancer Institute (NKI‐AvL) The Netherlands

**Keywords:** autoimmune disease, B‐cell activation and differentiation, B cells, PI3K‐AKT‐mTOR, plasma cells

## Abstract

Antibody production by the B cell compartment is a crucial part of the adaptive immune response. Dysregulated antibody production in the form of autoantibodies can cause autoimmune disease. To date, B‐cell depletion with anti‐CD20 antibodies is commonly applied in autoimmunity, but pre‐existing plasma cells are not eliminated in this way. Alternative ways of more selective inhibition of antibody production would add to the treatment of these autoimmune diseases. To explore novel therapeutic targets in signaling pathways essential for plasmablast formation and/or immunoglobulin production, we performed a compound screen of almost 200 protein kinase inhibitors in a robust B‐cell differentiation culture system. This study yielded 35 small cell‐permeable compounds with a reproducible inhibitory effect on B‐cell activation and plasmablast formation, among which was the clinically applied mammalian target of rapamycin (mTOR) inhibitor rapamycin. Two additional compounds targeting the phosphoinositide 3‐kinase‐AKT‐mTOR pathway (BKM120 and WYE‐354) did not affect proliferation and plasmablast formation, but specifically reduced the immunoglobulin production. With this compound screen we successfully applied a method to investigate therapeutic targets for B‐cell differentiation and identified compounds in the phosphoinositide 3‐kinase‐AKT‐mTOR pathway that could specifically inhibit immunoglobulin production only. These drugs may well be explored to be of value in current B‐cell‐depleting treatment regimens in autoimmune disorders.

## Graphical abstract

Through a large compound screen involving 192 inhibitors, the functional effects on B‐cell activation, differentiation, and antibody production were investigated. This highlighted multiple compounds, of which drugs targeting the phosphoinositide 3‐kinase‐AKT‐mTOR‐axis were further validated. These drugs could complement current B‐cell‐depleting treatment regimens in autoantibody‐mediated autoimmune disorders

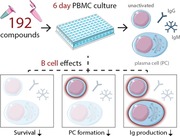

## Introduction

The B cell compartment is a fine‐tuned machinery to produce immunoglobulins protecting us from a variety of pathogens. However, antibodies can be harmful in several diseases where autoantibodies directed towards ‘self’ play a role in development of disease: e.g. systemic lupus erythematosus (SLE), rheumatoid arthritis, and primary immunodeficiency associated with autoimmune cytopenias 1, 2. More insight in which signaling pathways are essential for peripheral B‐cell differentiation and immunoglobulin production is needed, to identify which molecules can be interfered with to develop more specific B cell targeted therapies preventing autoantibody production.

We combined our previously published culture system 3 with the application frequently used in cancer research to test a large series of small cell‐permeable drugs in a compound screen by their ability to inhibit peripheral blood B cells to proliferate and differentiate into activated Immunoglobulin (Ig)‐secreting plasmablasts. Several libraries of well‐validated inhibitors of different cellular pathways are nowadays available. Compound, RNA interference (RNAi) and short hairpin RNA (shRNA) screens are frequently used in the field of cancer, looking for targets that specifically kill target cells 4, 5. For the purpose of this study, a different approach is needed to evaluate inhibition of any of the stages of peripheral B cell differentiation rather than just cell death. We were able to adapt our frequently used culture system into a robust, feasible, and easy read‐out system to perform a high‐throughput screen based on B‐cell activation and differentiation.

The aim of our compound screen was twofold. First, the results could lead to more detailed insight in signaling pathways not yet well described in peripheral B‐cell activation and differentiation involved in early plasma cell formation. Second, our findings could lead to the identification of potential drug targets for adjuvant treatment of autoantibody‐mediated disease.

The latter aim is becoming relevant because many autoimmune diseases are targeting B cells, as exemplified by current treatment regimens with anti‐CD20 antibodies for B‐cell depletion. Although plasmablasts are poised to become long‐lived immunoglobulin‐producing plasma cells, these are not well targeted by anti‐CD20 biologicals upon in vivo treatment, as they have lost most of their CD20 surface expression 6, 7. Evidence of the applicability of additional B cell‐specific small inhibitors is urgently needed to treat therapy‐resistant and clinically resilient conditions of autoimmunity that are often accompanied by increased morbidity and mortality.

In this study we applied a compound screen of almost 200 protein kinase inhibitors using a robust in vitro B‐cell differentiation culture system. This resulted in three compounds that have an interesting and B cell‐specific effect by blocking B‐cell differentiation into plasmablasts and/or immunoglobulin production. One of the compounds was the immunosuppressive drug rapamycin, a well‐defined mTORC1 inhibitor. Although data on T‐cell inhibition are well‐known, effects of rapamycin on human B‐cell activation and differentiation is only recently described by Traitanon et al. 8. The other two compounds are novel targets, interfering with the phosphoinositide 3‐kinase (PI3K)‐AKT‐mammalian target of rapamycin (mTOR) pathway similar to rapamycin, that inhibit immunoglobulin production by normally differentiated plasmablasts or short‐lived plasma cells at lower concentrations. These data hold great promise to prevent or treat antibody‐mediated disease in patients.

## Results

### B‐cell differentiation‐based decision model for compound screening

**Figure 1:**
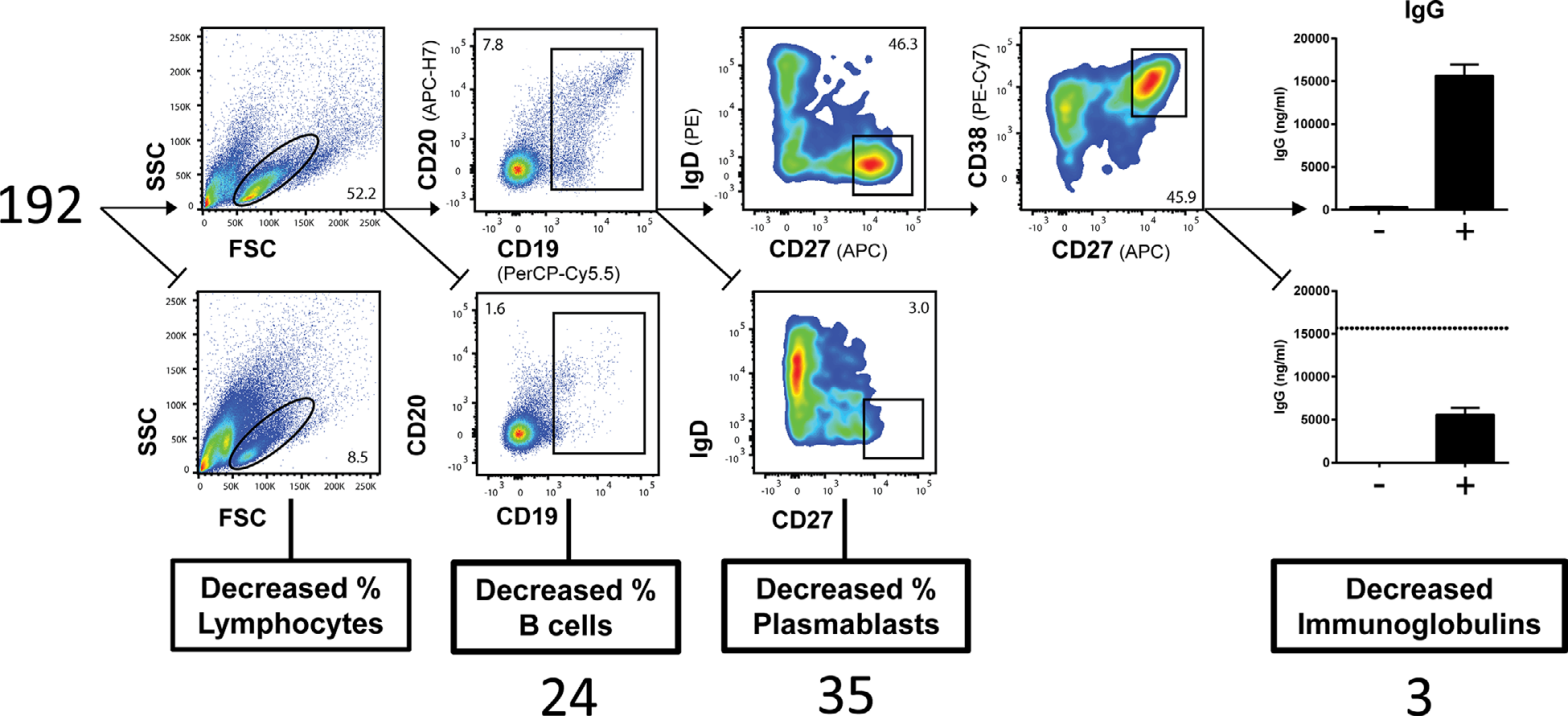
B‐cell differentiation‐based decision tree for compound screening. The gating strategy for the compound screen after 6 days of PBMC culture with CpG/IL‐2 stimulation: from left to right, each respective compound was classified based on percentage of lymphocytes gated by forward scatter (FSC)/side scatter (SSC); percentage of CD19^+^ CD20^+^ B cells; percentage of CD27^++^ CD38^++^ plasmablasts; and IgG, IgM, and IgA production (only a reduction in all immunoglobulins was classified as ‘Decreased Immunoglobulins’). Cut‐off values can be found in the *Materials and Methods*, pooled data (*n* = 3) from three independent experiments.

For high‐throughput compound screening we adapted our previously published assay on B cell‐activation and differentiation during a 6‐day B cell culture 3 to a user‐friendly 96‐well plate format and automated plate‐reading by flow cytometry, using standardized gating and assessment strategy. In the presence of a low concentration of IL‐2, B cells were activated with CpG as a classical toll‐like receptor 9 (TLR9) ligand to stimulate B cells directly in a B cell antigen receptor‐independent way 3, 9. After 6 days of CpG stimulation, B cells differentiated into immunoglobulin‐producing cells, demonstrated by the appearance of activated plasmablasts (sIgD^−^/CD27^++^/CD38^++^) and the production of immunoglobulins IgG, IgM, and IgA in the culture supernatants (Fig. 1).

To assess which of the protein kinase‐inhibitors had a strong and reproducible effect on B cell function and Ig production, a decision‐tree was made to select the compounds that were of interest for further testing. Compounds reducing percentages of lymphocytes were discarded, assuming those inhibitors induced more generalized cell death. Using our decision tree and criteria we selected 62 compounds of potential interest (Supporting Information Table 1). Of these 62 compounds, 24 compounds induced B cell death or reduced B cell proliferation as indicated by the reduced B cell percentage, 35 decreased CD27^++^CD38^++^ plasmablast formation, and three left plasmablast formation intact but impaired the immunoglobulin production for all isotypes (IgG, IgM, IgA) during the 6‐day culture (Fig. 1). The 38 compounds that did not affect B cell survival were selected for further study and included compounds inhibiting kinases of the PI3K‐Akt‐mTOR pathway (nine compounds), MAPK pathway (9), angiogenesis pathway (7), RTK pathway (7), cell‐cycle pathway (4), and JAK‐STAT pathway (2).

### Validation of compounds inhibiting plasmablast formation

First, the 35 compounds that inhibited plasmablast formation in the initial screen were tested in a follow‐up experiment. Different concentrations (10^−3^–10^1^ µM) were used around the originally used dose of 1 µM to study dose‐dependent effects of the compounds on B‐cell differentiation and plasmablast‐dependent immunoglobulin production (Supporting Information Fig. 2).

**Figure 2:**
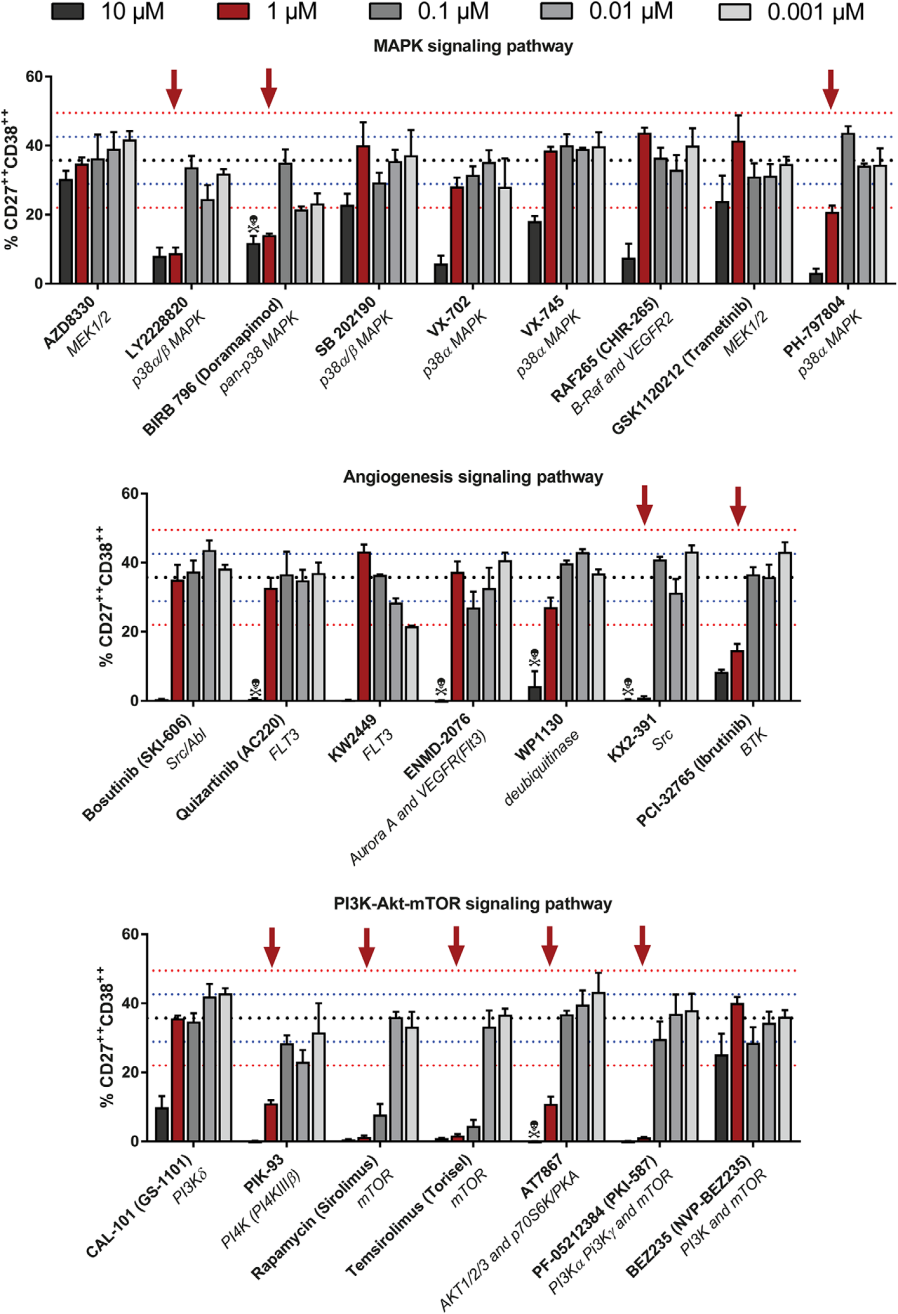
Validation of plasmablast‐inhibiting compounds. Plasmablast‐inhibiting compounds of the initial screening were validated in multiple concentrations around the initial dose of 1 µM. Again, PBMCs were stimulated with CpG/IL‐2 for 6 days. Plasmablasts were gated as CD19^+^CD20^dim/+^CD27^++^CD38^++^. Shown are the MAPK, angiogenesis, and PI3K‐AKT‐mTOR signaling pathways. Pooled data (*n* = 3) from three independent experiments. *Black dotted line*: mean percentage of CD27^++^ CD38^++^ B cells after 6 days of CpG/IL‐2 stimulation without compound (*n* = 72). *Blue dotted line*: ±1 SD. *Red dotted line*: ±2 SD. *Red arrow*: percentage of CD27^++^CD38^++^ B cells below −2 SD of CpG/IL‐2 stimulated cells without compound at the concentration used in the initial screen (1 µM). *Toxicity symbol*: percentage of lymphocytes below −2 SD of CpG/IL‐2 stimulated cells without compound.

Out of these 35 compounds initially selected, 24 showed a reproducible plasmablast‐inhibiting effect at 1 µM, however, only 11 showed a very strong reduction in CD27 and CD38 upregulation at that concentration (defined as ≥‐2SD of the mean % CD27^++^CD38^++^ of stimulated cells without compound). This highlighted three pathways with compounds that showed the most potent inhibiting effects on plasmablast differentiation; the PI3K‐AKT‐mTOR signaling pathway, the MAPK signaling pathway, and the Angiogenesis signaling pathway (Fig. 2). The compounds interfering with the MAPK signaling pathway all inhibited the kinase p38α, three out of six showing a two to fourfold reduction of plasmablast formation at 1 µM. BTK inhibitor PCl‐32765, also known as Ibrutinib, and KX2‐391 (Src inhibitor) were two potent inhibitors originally classified as angiogenesis signaling pathway inhibitors. Clearly, most effective inhibitors of plasmablast formation were compounds interfering in the PI3K‐AKT‐mTOR pathway. PIK‐93, AT7867, and PF‐05212384 all showed plasmablast inhibition, although AT7867 induced toxic effects on all lymphocytes at the highest concentration.

Rapamycin and its derivative temsirolimus were the strongest inhibitors in this set, reducing plasmablast formation at 0.1 µM. Since rapamycin is widely applied as an immunosuppressive drug in the transplantation setting, and because its applicability in autoimmune diseases is being explored, we investigated its specific B cell suppressive property.

### B cell specific effects of mTOR inhibitor rapamycin on B‐cell differentiation

The effects of rapamycin on human B cell function have not been characterized in great detail 10, 11, 12. We used rapamycin in the therapeutic dose range (in ng/mL), which was lower than the concentration of the initial compound screen. We compared rapamycin to concentrations of additional immunosuppressive drugs that are currently used to treat autoimmune disease or are being applied in clinical transplantation settings.

**Figure 3:**
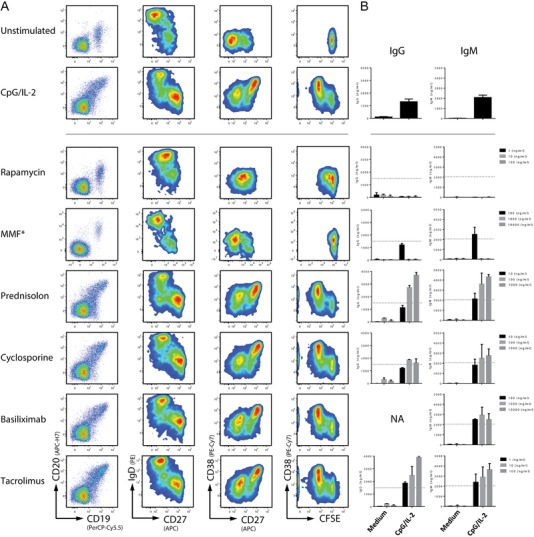
B cell effects of rapamycin compared to commonly applied immunosuppressive drugs. (A) Flow cytometry plots for B‐cell proliferation by CFSE dilution and differentiation into plasmablast (IgD^−^/CD27^++^/CD38^++^) after 6 days of culture with CpG/IL‐2. Therapeutic range concentrations were used for every immunosuppressive drug, including a tenfold higher and lower concentration. Shown are representative plots of the middle, therapeutic range, concentration of multiple experiments (*n* = 3). *Asterisk*: representative plots for mycophenolate mofetil (MMF) are from a separate experiment, since the three initially used concentrations were all lower than the therapeutic dose range. Now the correct concentration for MMF is shown, 1.000 ng/mL. (B) ELISA of IgG and IgM in the supernatant after 6 days of B cell culture at three concentrations for every immunosuppressive drug. Multiple experiments pooled and plotted as mean + SEM (*n* = 3 per concentration), dotted line equals mean of stimulated controls without any immunosuppressive drug added (*n* = 15).

Besides the mTOR inhibitor rapamycin, none of the clinically approved drugs had any significant effect on B cell proliferation and differentiation after TLR9 stimulation, except for mycophenolate mofetil (MMF), which is a known inhibitor of B and T cell proliferation 13, 14 (Fig. 3A). Rapamycin showed an inhibitory effect on plasmablast formation at therapeutic concentrations, as indicated by the diminished appearance of sIgD^−^/CD27^++^/CD38^++^ cells in our cultures. The production of IgG and IgM was completely aborted, as expected from the observed block in plasmablast formation (Fig. 3B). Notably, none of the other clinical‐grade immunosuppressive drugs (except MMF) that were tested in parallel decreased the in vitro immunoglobulin production, while an unexpected increase in immunoglobulin production was even present upon addition of prednisolone and tacrolimus. To further extend the value of the data on mTOR inhibition in human B cells, the effect of rapamycin was also found to be reproducible when stimulating B cells with a combination of anti‐IgM/anti‐CD40/IL‐21 as a condition that mimics T cell‐dependent B‐cell activation (data not shown). These findings support the idea that mTOR inhibition results in a general B cell‐intrinsic effect and not a stimulus‐specific blockade in intracellular signaling.

Unlike B cells, the effects of rapamycin on T cells were less prominent in the therapeutic dose range. The percentage of T cells dividing at least once was largely unaltered (data not shown). Expression of the activation markers CD25 and CD38 was not affected at any of the concentrations, and there were only minor shifts in the cytokine production (less IFN‐γ and IL‐17 in the supernatant of the cultures) (Supporting Information Fig. 2). Although minor inhibiting effects of rapamycin on T cells were seen, our data show that at therapeutic dose ranges B cells function are more drastically affected.

### BKM120 and WYE‐354 specifically reduce immunoglobulin production at lower concentrations

**Figure 4:**
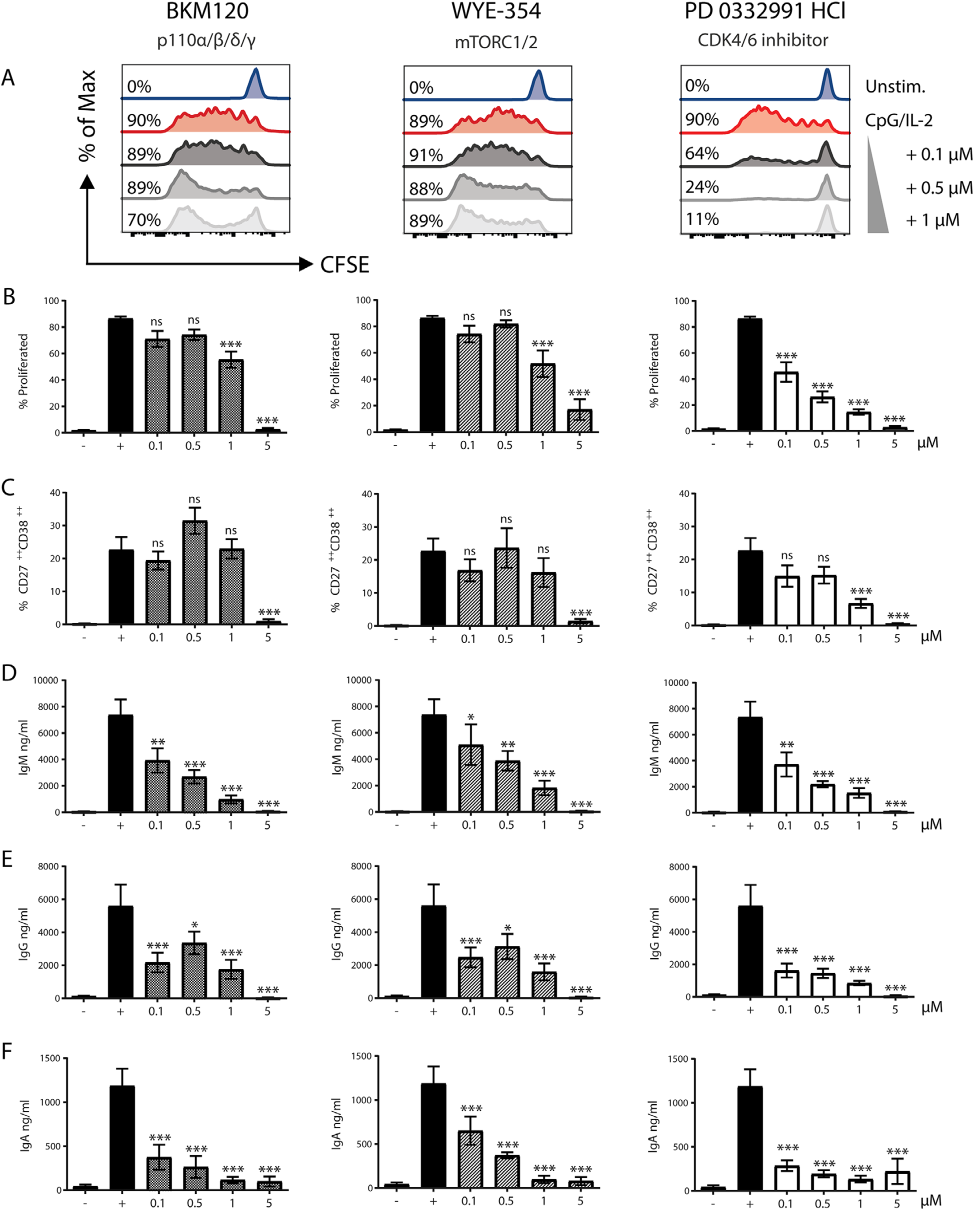
BKM120 and WYE‐354 reduce immunoglobulin production of all isotypes, without reducing plasmablast formation. Proliferation, plasmablast differentiation and immunoglobulin production for BKM120 (left), WYE‐354 (middle), and PD 0332991 (right) after 6 days of CpG/IL‐2 stimulation. (A) Representative histogram plots of CFSE for concentration 0.1–1 µM, percentages represent proportion B cells with at least one cell division. Summary graphs of multiple flow cytometry experiments for (B) percentage proliferated B cells and (C) percentage CD27^++^ CD38^++^ plasmablasts. Immunoglobulin production measured by ELISA in 6‐day culture supernatant for (D) IgM, (E) IgG, and (F) IgA. Symbols: − unstimulated, *+* stimulated with CpG/IL‐2. Multiple experiments pooled and shown as mean ± SEM (for concentrations 0.1–1 µM *n =* 6 to 10, concentration 5 µM *n* = 4). *p* values were determined by one‐way ANOVA, *ns* not significant, *p ≤ 0.05, **p ≤ 0.01, ***p ≤ 0.001.

Three compounds showed reduced immunoglobulin production while the plasmablast formation stayed intact: BKM120 (p110α/β/δ/γ‐inhibitor, also known as Buparlisib), WYE‐354 (ATP‐competitive inhibitor of mTORC1/2), and PD 0332991 (CDK4/6 inhibitor). Using CFSE dilution as a read‐out, we observed a clear dose‐dependent inhibition of the CpG‐induced proliferative response by adding the compound PD 0332991 (Fig. 4A and B). BKM120 and WYE‐354 showed no significant decrease in proliferation at concentrations up to 1 µM. There was a clear reduction at the highest concentrations (5 µM). Both plasmablast formation and immunoglobulin production were impaired at those high proliferation‐inhibiting concentrations for all three of the compounds as well. In the absence of an effect on proliferation, there was also no effect on plasmablast formation for both BKM120 and WYE‐354 at the lowest concentrations (Fig. 4C), whereas a clear (dose‐dependent for IgM and IgA) suppressive effect on immunoglobulin production was observed (Fig. 4D–F).

This effect was independent of the type of stimulation, since the same pattern of reduced IgG production was observed upon stimulation with anti‐IgM/anti‐CD40/IL‐21(Supporting Information Fig. 3). Next, we excluded cytotoxicity effects at lower concentrations, yet a drop in percentage of live cells started to appear at 1 µM in all lymphocytes (Supporting Information Fig. 4). Functional effects on T‐cell activation started only at the concentration of 5 µM, mainly resulting in impaired proliferation and CD38 upregulation when BKM120 was added (Supporting Information Fig. 5).

Overall, PD 0332991 seemed to reduce immunoglobulin production by inhibiting proliferation as expected from its inhibition of cell‐cycle by targeting CDK4/6, while both compounds interfering in the PI3K‐Akt‐mTOR pathway, i.e. BKM120 and WYE‐354, showed more specific ‘immunoglobulin‐inhibiting only’ features.

To verify whether other PI3K‐inhibitors had the same effect as BKM120, we tested a currently clinically applied drug that was initially classified as ‘inhibiting plasmablast formation’ in our high throughput screen (Fig. 1 and Table 1): CAL‐101 (GS‐1101), also known as Idelalisib, a clinically approved drug targeting p110δ. By validating these results, we distinguished plasmablast inhibitory effects similar to rapamycin at higher concentrations (≥1µM, Fig. 2) from effects on immunoglobulin production only, identical to BKM120 and WYE‐354 at lower concentrations (≤1µM, Supporting Information Fig. 6), hereby confirming similar characteristics of other drugs that interfere with PI3K‐AKT‐mTOR signaling.

**Table 1:** List of 62 compounds selected according to the decision tree. Compound name, protein kinase target (Kinase Inhibitor Library, catalog no. L120, from Selleck Chemicals) and corresponding signaling pathway involved are being indicated. We found 24 compounds that affected B cell survival and/or proliferation, 35 that showed a decrease in plasmablast formation and immunoglobulin production (decreased CD27 and CD38 upregulation) and three that had no clear impact on plasmablast formation but impaired in vitro immunoglobulin release (reduction in all immunoglobulins; IgM, IgG, and IgA) upon activation and culture with CpG/IL‐2

Decreased % of B cells			Decreased % of Plasmablasts			Decreased Ig production		
Compound name	Inhibits	Pathway	Compound name	Inhibits	Pathway	Compound name	Inhibits	Pathway
Ponatinib (AP24534)	Abl	Angiogenesis signaling	ENMD‐2076	Aurora A and VEGFR(Flt3)	Angiogenesis signaling	PD 0332991 HCl	CDK4/6	Cell‐cycle signaling
Hesperadin	Aurora B	Cell‐cycle signaling	PCI‐32765 (Ibrutinib)	BTK	Angiogenesis signaling	BKM120 (NVP‐BKM120)	p110α/β/δ/γ	PI3K‐AKT‐mTOR signaling
MLN8237 (Alisertib)	Aurora A	Cell‐cycle signaling	WP1130	deubiquitinase	Angiogenesis signaling	WYE‐354	mTOR	PI3K‐AKT‐mTOR signaling
VX‐680 (MK‐0457, Tozasertib)	Aurora A	Cell‐cycle signaling	KW 2449	FLT3	Angiogenesis signaling			
PHA‐680632	Aurora A/B/C	Cell‐cycle signaling	Quizartinib (AC220)	FLT3	Angiogenesis signaling			
GSK461364	Plk1	Cell‐cycle signaling	KX2‐391	Src	Angiogenesis signaling			
Danusertib (PHA‐739358)	Aurora A/B/C	Cell‐cycle signaling	Bosutinib (SKI‐606)	Src/Abl	Angiogenesis signaling			
SNS‐314	Aurora A/B/C	Cell‐cycle signaling	ZM‐447439	Aurora A and Aurora B	Cell‐cycle signaling			
CYC116	Aurora A/B	Cell‐cycle signaling	Aurora A Inhibitor I	Aurora A	Cell‐cycle signaling			
HMN‐214	Plk1	Cell‐cycle signaling	LY2603618 (IC‐83)	Chk1	Cell‐cycle signaling			
Barasertib (AZD1152‐HQPA)	Aurora B	Cell‐cycle signaling	LY2784544	JAK2	JAK‐STAT signaling			
ON‐01910	Plk1	Cell‐cycle signaling	Tofacitinib citrate (CP‐690550 citrate)	JAK3	JAK‐STAT signaling			
TAK‐901	Aurora A/B	Cell‐cycle signaling	RAF265 (CHIR‐265)	B‐Raf and VEGFR2	MAPK signaling			
AMG 900	Aurora A/B/C	Cell‐cycle signaling	VX‐702	p38α MAPK	MAPK signaling			
AT9283	JAK2/3	JAK‐STAT signaling	AZD8330	MEK1/2	MAPK signaling			
AZ 960	JAK2	JAK‐STAT signaling	GSK1120212 (Trametinib)	MEK1/2	MAPK signaling			
Cyt387	JAK1/JAK2	JAK‐STAT signaling	LY2228820	p38α/β MAPK	MAPK signaling			
Deforolimus (Ridaforolimus)	mTOR	PI3K‐AKT‐mTOR signaling	PH‐797804	p38α MAPK	MAPK signaling			
WYE‐125132	mTOR	PI3K‐AKT‐mTOR signaling	VX‐745	p38α MAPK	MAPK signaling			
Ku‐0063794	mTORC1 and mTORC2	PI3K‐AKT‐mTOR signaling	SB 202190	p38α/β MAPK	MAPK signaling			
PP242	mTOR	PI3K‐AKT‐mTOR signaling	BIRB 796 (Doramapimod)	pan‐p38 MAPK	MAPK signaling			
ZSTK474	PI3K	PI3K‐AKT‐mTOR signaling	AT7867	AKT1/2/3 and p70S6K/PKA	PI3K‐AKT‐mTOR signaling			
GDC‐0941	PI3K	PI3K‐AKT‐mTOR signaling	Rapamycin (Sirolimus)	mTOR	PI3K‐AKT‐mTOR signaling			
AZD8055	mTOR	PI3K‐AKT‐mTOR signaling	Temsirolimus (Torisel)	mTOR	PI3K‐AKT‐mTOR signaling			
			BEZ235 (NVP‐BEZ235)	PI3K and mTOR	PI3K‐AKT‐mTOR signaling			
			CAL‐101 (GS‐1101)	PI3Kδ	PI3K‐AKT‐mTOR signaling			
			PF‐05212384 (PKI‐587)	PI3Kα, PI3Kγ and mTOR	PI3K‐AKT‐mTOR signaling			
			PIK‐93	PI4K (PI4KIIIβ)	PI3K‐AKT‐mTOR signaling			
			TAE684 (NVP‐TAE684)	ALK	RTK signaling			
			MGCD‐265	c‐Met and VEGFR1/2/3	RTK signaling			
			WZ3146	EGFR	RTK signaling			
			WZ8040	EGFR	RTK signaling			
			Mubritinib (TAK 165)	HER2/ErbB2	RTK signaling			
			Foretinib (GSK1363089, L880)	Met and KDR	RTK signaling			
			PP‐121	PDGFR, Hck, mTOR, VEGFR2, Src and Abl	RTK signaling			

### Reduction in immunoglobulin‐production by plasmablasts related to strength of mTOR‐inhibition

**Figure 5:**
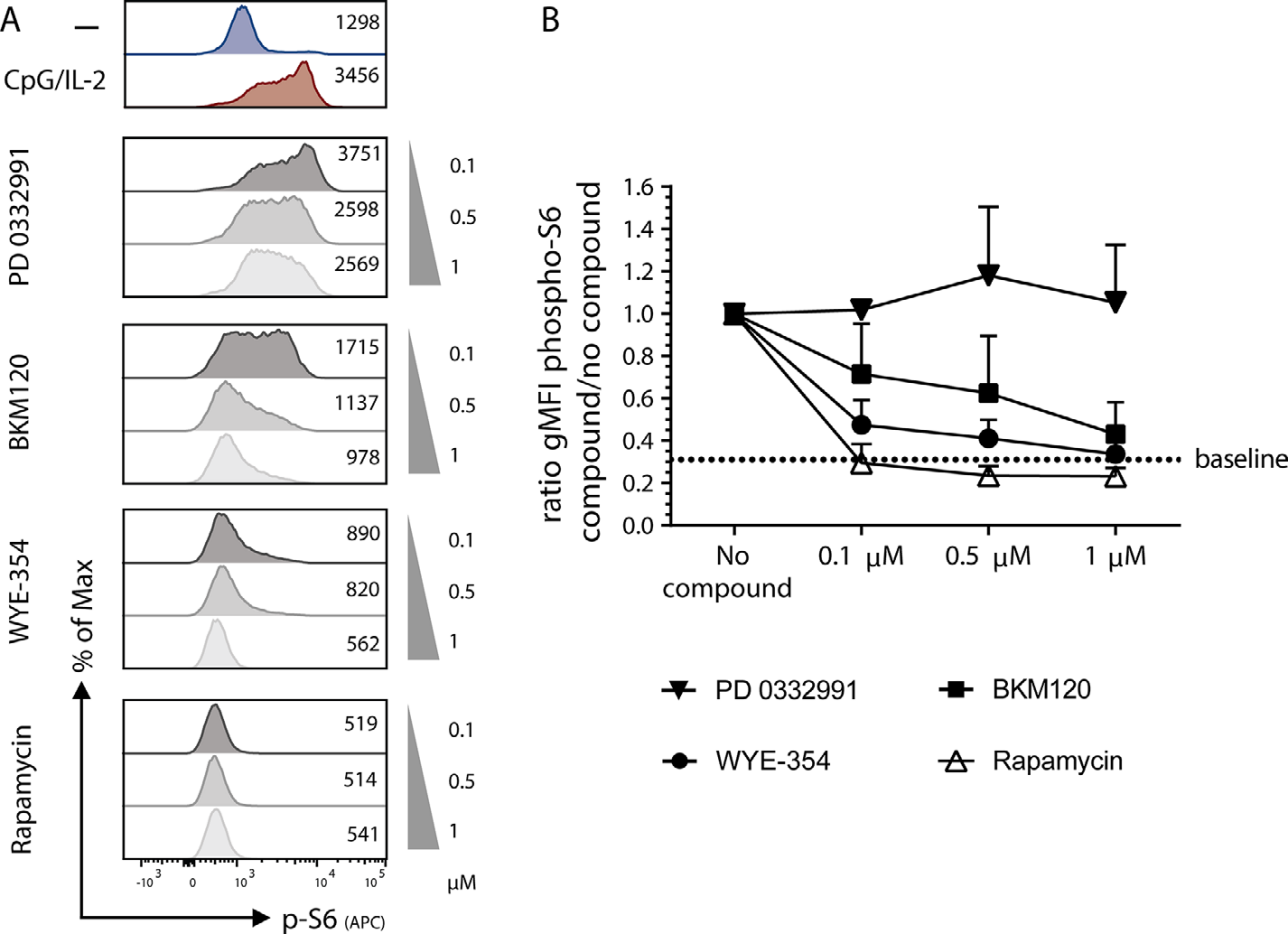
Partial inhibition of pospho‐S6 upon activation by adding BKM120 and WYE‐354. PBMCs were stimulated for 4 h with CpG/IL‐2 and different concentrations of compound were added, 0.1–1 µM. Phosphorylated‐S6 (gMFI) was measured as a read‐out for mTORC1 activation, gated on CD19^+^ B cells. (A) Representative histogram plots of p‐S6 after stimulation with increasing amounts of compounds with rapamycin as a control, numbers represent gMFI. (B) Pooled summary graph of multiple experiments (mean + SEM, *n* = 3), the ratio p‐S6 expression after CpG/IL‐2 with the addition of a compound at a certain concentration divided by the p‐S6 expression without compound. Dotted line labeled as ‘baseline’ indicates the mean ratio of the unstimulated samples without compound.

To examine the specificity of those PI3K‐AKT‐mTOR inhibiting compounds and narrow down on the inhibition of mTORC1 as a cause of the reduction in immunoglobulin production only, we used an intracellular staining of phosphorylated S6 ribosomal protein (pospho‐S6) as a read‐out of mTORC1 activation after 4 h of CpG/IL‐2 stimulation (Fig. 5A). The addition of rapamycin led to the absence of an increase in phospho‐S6 upon stimulation in B cells as expected, even at the lowest concentrations. In accordance with the effects seen at a functional level, there was a difference in phospho‐S6 inhibition for both BKM120 and WYE‐354 depending on the concentration used (Fig. 5A and B). At the concentration of 5 µM, the phospho‐S6 expression was found to be as low as any concentration of rapamycin. However, at lower concentrations an intermediate reduction was observed.

These data suggest that partial inhibition of the PI3K‐AKT‐mTOR pathway might lead to a reduction of immunoglobulin production only, while full inhibition leads to inhibition of proliferation and plasmablast formation with subsequent inhibition of immunoglobulin production.

## Discussion

In a screen of small molecules with known kinase‐inhibiting characteristics we have tested the effects of almost 200 cell‐permeable compounds on B cell survival, proliferation, differentiation, and immunoglobulin production. The role of the PI3K‐AKT‐mTOR pathway in human B cell plasmablast differentiation 10, 12, 15 was supported by the identification of drugs in our in vitro compound screen for adjuvant treatment of autoantibody‐mediated disease.

Screening of the library of kinase inhibitors yielded 24 compounds that reduced B cell outgrowth and 38 small molecules were identified to inhibit end‐stage plasmablast differentiation. The first group of compounds, inhibiting B cell survival and/or proliferation, mainly comprised cell‐cycle signaling inhibitors as expected. Despite being of interest for the field of cancer and other lymphoproliferative disorders, we focused on the more advanced stages of plasmablast differentiation in this study. Hence, the second category of 35 compounds inhibiting end‐stage differentiation by reducing plasmablast formation and as a consequence an absence of immunoglobulin production was considered more relevant.

Most compounds that showed inhibition of plasmablast differentiation were targeting the PI3K‐AKT‐mTOR pathway, being inhibitors of either early (PIK‐93, targeting PI4KIIIβ) or late (rapamycin, temsirolimus, both targeting mTORC1) signaling components in this pathway. While focusing on this more prominent inhibition of PI3K and mTOR, we additionally observed that inhibition of p38α, part of the MAPK signaling pathway, blocked plasmablast formation in multiple compounds targeting this kinase. To date, not much is known about the role of p38α (MAPK14) in B cells. It was described that p38α is dispensable for lymphocyte proliferation in mice 16, but no data on human plasmablast differentiation are available. Also, PCI‐32765, a BTK inhibitor used in clinical practice in the treatment of CLL as Ibrutinib, has significant effects on B‐cell differentiation. However, Ibrutinib can inhibit T‐cell activation as well by inhibiting ITK 17, which might be responsible for the occurrence of opportunistic infections in treated patients 18. Indeed, in our assays Ibrutinib showed to be not strictly B cell selective demonstrating significant impact on T‐cell function (data not shown).

In the kinase library we used for this screen, targeting the PI3K‐AKT‐mTOR axis resulted in a variety of outcomes, from inducing generalized lymphocyte death to no effect at all. The kinase mTOR is downstream of the PI3K‐AKT pathway and plays an important role in several cellular functions involving cell growth, proliferation, and angiogenesis 19. A limited number of publications is available that describe mTOR regulation of B cell immunity and differentiation, mostly in mouse studies. In mice, B cells with a strongly reduced mTOR activity show decreased differentiation into plasma cells in response to antigen stimulation in an experimental mTOR‐hypomorphic model 20. A complete deletion of the mTOR inhibitory molecule TSC‐1, leading to hyperactive mTOR signaling also results in defects in plasma cell differentiation after immunization 21. Although not fully explained, these studies suggest that both high and low mTOR activity can lead to suboptimal conditions of plasma cell differentiation.

The outcomes of using PI3K or mTOR inhibitors in mice vary widely. Limon et al. showed in mouse models that instead of inhibiting B cell proliferation and differentiation at high doses, class switch recombination can be enhanced at low concentrations of ATP‐competitive mTOR inhibitors 22. It was hypothesized that this is caused by mTORC2‐inhibiting effects being dominant over mTORC1‐inhibition at submaximal concentrations, leading to increased IgG1 class switching. In their mouse studies PI3K or AKT inhibitors showed similar effects, since FoxO transcription factor repression by AKT is lost. In contrast, other groups reported reduced class switch recombination by limiting mTOR signaling, which might be due to differences in efficiency of mTORC1 and/or mTORC2 suppression 23, 24. Together these experimental mouse studies confirm there is a delicate balance in PI3K‐AKT‐mTOR signaling, and subtle variations in dose, target, and binding affinity of the inhibitor can have differential effects on B‐cell activation, differentiation, and antibody production, essentially as we describe here.

Apart from the abovementioned mouse studies, certain defects in the activation of the PI3K/AKT/mTOR pathway may also influence B cell development or differentiation in human. Little was known about the clinical consequences of any defect in these signaling cascades until constitutively active PI3K signaling was described in Activated PI3Kδ Syndrome (APDS) patients. In APDS, a combined B‐ and T‐cell dysfunction is caused by *PIK3CD* or *PIK3R1* gene mutations 25, 26, 27. The dysimmunoglobulinemia may consist of an increase in IgM and decrease in IgG or IgG‐subclass levels. Studies on patient‐derived T lymphocytes showed that the mutations in APDS cause increased phosphorylation of AKT and hyperphosphorylation of the downstream mTOR signaling pathway, with increased glucose usage in the cells and predisposition to differentiation and concomitant senescence.

The opposite was described in patients with p110δ loss‐of‐function in two separate families 28, 29, clinically suffering from hypogammaglobulinemia and poor response upon immunization. There are no cases identified thus far in which a genetic defect results in an AKT or mTOR loss (or gain) of function. The findings reported on p110δ deficiencies confirm our study, emphasizing the essential role of adequate mTOR signaling in human plasmablast formation and immunoglobulin production.

Preliminary data on clinical treatment with mTOR inhibitors of patients with a common autoimmune disease such as SLE show promising results 30, as well as a more recent cohort study on the treatment of autoimmune cytopenias 31. Although most effects of mTOR inhibition have been suggested to result from T‐cell inhibition, the effects on B cells have not yet been well investigated except for one recent study 8 showing similar results as we describe here. If sufficient in vivo evidence is obtained to demonstrate a block in plasma cell induction and/or survival, mTOR inhibitors should be considered for the ability to treat severe allo‐ or autoantibody‐mediated disease conditions at relatively low doses that may leave normal T cell immunity intact and thus avoid many of the more severe side effects of T‐cell immunosuppression.

In addition to rapamycin, we characterized two other drugs that target B cells by interfering with the PI3K‐AKT‐mTOR pathway, BKM120 (Buparlisib) and WYE‐354. We showed that both were unique in their ability to reduce immunoglobulin production without affecting proliferation and plasmablast formation at concentrations considered relevant to clinical application. Although the immunoglobulin production was not reduced to complete absence, this is a feature that can be of interest for diseases where autoantibodies play a role, such as rheumatoid arthritis, SLE, and autoimmune cytopenias. Currently, patients with autoantibody mediated diseases are commonly treated with anti‐CD20 to eradicate all circulating B cells. However, it has been well described that anti‐CD20 regimens do not efficiently eradicate all memory B cell pools 7, 32, which might explain residual disease activity, relapse, or therapy‐resistance in autoimmune diseases. The adjuvant use of PI3K and mTOR inhibitors in these cases has been hypothesized for a long time and underpinned by mouse studies 12, 33 and the aforementioned p110δ loss‐of‐function immunodeficiencies 28, 29. Treatment with rapamycin in a murine SLE model reversed disease by reducing production of both allo‐ and autoantibodies 34. This effect of in vivo inhibition of mTORC1 was attributed to a block in newly formed plasma cells complemented by diminished antibody secretion by the long‐lived plasma cells that still were surviving, showing these two processes can be uncoupled.

This is now complemented by the human in vitro data we describe here on BKM120, WYE‐354, and Idelalisib. The first is currently tested in clinical trials to treat solid tumors and appears to be safe and well tolerated 35, the latter being clinically available already to treat certain hematological malignancies in combination with anti‐CD20 therapy 36, 37. Taken together, the drugs highlighted in this study might add to the treatment regimen of a variety of autoimmune diseases by inhibiting either end‐stage plasmablast differentiation (rapamycin) or immunoglobulin production only (BKM120, WYE‐354, Idelalisib).

In sum, we successfully screened a library of small molecule kinase inhibitors for their potential therapeutic effect on human B cell survival, proliferation, differentiation, and immunoglobulin production. Our study clearly demonstrates inhibitory effects of the mTOR inhibitor rapamycin and an interesting immunoglobulin‐inhibiting effect of two additional PI3K‐AKT‐mTOR inhibitors. Besides its function in general cell homeostasis, mTOR is critical for plasmablast formation and immunoglobulin production. The level of inhibition may even separate plasmablast formation from immunoglobulin production itself, which offers an interesting lead to further explore more selective drug targeting effects. PI3K‐AKT‐mTOR inhibition might add to the treatment of autoimmune diseases where autoantibodies produced by B cells play a role and could complement treatment with B cell depletion or when anti‐CD20 regimens show insufficient effectiveness.

## Materials and Methods

### Samples

The study was approved by Medical Ethics Committee of the Academic Medical Center (NL40331.078.12) in accordance with the Declaration of Helsinki. We obtained PBMCs from buffy coats from healthy donors. PBMCs were isolated according to standard density gradient centrifugation using Lymphoprep (Nycomed, Oslo, Norway), and stored in liquid nitrogen until use.

### Flow cytometry

PBMCs were resuspended in PBS, containing 0.5% w/v BSA and 0.01% sodium azide. PBMCs were incubated with saturating concentrations of fluorescently labeled conjugated mAbs. Analysis of cells was performed using a FACSCanto‐II flowcytometer and FlowJo software (version 9.1 and 10) and for the methods of flow cytometry we adhered to the ‘Guidelines for the use of flow cytometry and cell sorting in immunological studies’ 38. The following mAbs were used for flow cytometry: CD3 Alexa Fluor 700 [557943], CD4 PE‐Cy7 [348809], CD8 PerCP‐Cy5.5 [341050], CD19 Alexa Fluor 700 [557921], CD19 PerCP‐Cy 5.5 [332780], CD20 APC‐H7 [641414], CD20 PerCP‐Cy5.5 [332781], CD25 APC [340907], CD27 APC [337169], CD38 PE [345806], CD38 PE‐Cy7 [335825], HLA‐DR FITC [347400], and IgD PE [555779] from BD (San Jose, USA); CD3 Alexa 700 [56‐0038‐41], CD19 Alexa Fluor 700 [56‐0199‐42], and CD27 APC‐eFluor 780 [47‐0279‐42] from eBioscience (San Diego, USA); and CD27 FITC [M1764] from Sanquin (Amsterdam, the Netherlands). To assess lymphocyte viability TO‐PRO‐3 iodide [T3605] was used (Thermo Fisher Scientific, Massachusetts, USA).

### B‐cell and T‐cell activation in vitro

Cells were washed and subsequently resuspended in IMDM supplemented with 10% fetal calf serum (BioWhittaker), antibiotics, and 3.57 × 10^–4^% v/v β‐mercaptoethanol (Merck). PBMCs were plated containing a fixed number of B cells (10^4^ per well) and were cultured in 96‐well flat‐bottomed plates for 6 days at 37°C and stimulated with 1 µg/mL CpG oligodeoxynucleotide 2006 (Invivogen), with 100 U/mL IL‐2 (R&D Systems), or anti‐IgM mAb (clone MH15; Sanquin), with anti‐CD40 mAb (clone 14G7; Sanquin) and 20 ng/mL IL‐21 (Invitrogen). For T‐cell stimulation, saturating amounts of aCD3 (clone 1xE) and aCD28 (clone 15E8) were added and cells were cultured for 3 days at 37°C.

### Intracellular phosphorylated‐S6 staining

Extracellular staining as described was followed by fixation and permeabilization using reagents of eBioscience (fixation/permeabilization concentrate [00‐5123‐43], fixation/permeabilization diluent [00‐5223‐56], 10x permeabilization buffer [00‐8333‐5] diluted by distilled water), subsequently stained by the primary antibody pospho‐S6 ribosomal protein Ser240/244 (D68F8), XP® rabbit (Cell Signaling), and a secondary goat anti‐rabbit IgG1 F(ab)2 APC‐conjugated antibody (Santa Cruz, SC‐3846). Analysis was performed with FACSCanto‐II flowcytometer and FlowJo software as described.

### ELISA

IgM, IgG, and IgA in supernatants were tested with an in‐house ELISA using polyclonal rabbit anti‐human IgM, IgG, IgA reagents and a human serum protein calibrator all from Dako (Heverlee, Belgium), as described before 3. In the T cell experiments, cytokine production was tested in the culture supernatants using eBioscience´s ELISA kits for IFN‐γ (88‐7316‐88), IL‐13 (88‐7439‐88), and IL‐17 (88‐7176‐88). ELISA was performed according to the manufacturer's instructions.

### Validation compounds of interest

In addition to the assessment of B‐ and T‐cell activation as described, PBMCs were labeled with 0.5 µM CFSE (Molecular Probes) for cell proliferation analysis. The following clinically applied immunosuppressive drugs were used to compare with rapamycin (Wyeth laboratories, Philadelphia, USA; 1, 10, and 100 ng/mL): tacrolimus (Fujisawa Holland BV, Houten, The Netherlands; 1, 10, and 100 ng/mL), basaliximab (Novartis Pharma AG, Basel, Switzerland; 100, 1000, and 10 000 ng/mL), cyclosporin A (Novartis Pharma AG, Basel, Switzerland; 10, 100, and 1000 ng/mL), prednisolon (ACE pharmaceuticals, Zeewolde, The Netherlands; 10, 100, and 1000 ng/mL), MMF (Roche, New Jersey, USA; 1, 10, 100, 1000, and 10 000 ng/mL), rapamycin (Wyeth laboratories, Philadelphia, USA; 1, 10, 100 ng/mL). Note that IgG levels cannot be measured when Basiliximab is used. From the initial screen, three compounds that showed interesting effects on immunoglobulin production only, were further tested: PD 0332991 HCL (Sigma‐Aldrich, Missouri, USA), BKM120 (NVP‐BKM120, Buparlisib) (Selleck Chemicals, Texas, USA), WYE‐354 (Cayman Chemical, Michigan, USA).

### High throughput compound screen sampling and plating

All liquid handling steps were performed on the Hamilton STAR (Hamilton Company). Cells were seeded in screening plates using the Thermo Multidrop Combi (Thermoscientific). Compound dilutions were added to screening plates using the Hamilton STAR. All 192 compounds were purchased from the Kinase Inhibitor Library (Catalog no. L1200) from Selleck Chemicals (Houston, Texas) (see Supporting Information Table 1 for a full list of the compounds used) and were randomly distributed on 96‐well plates (96‐well cell culture plate, Corning Incorporated, Kennebunk, USA), with an initial dose of 1 µM. Compounds were plated at different positions per plate (*n* = 3, per compound) to avoid any spurious results due to zoning or potential effects of bias. On every plate, eight negative control wells (unstimulated, no compound/DMSO control) and eight positive control wells (stimulated with CpG/IL‐2, no compound/DMSO control) were added.

### Screening set‐up and cut‐off statistics

Measured values were plate‐normalized by dividing them by the mean of the positive controls (*n =* 72). A significant decrease in percentage for any of the analyzed populations was defined by the use of two criteria. The first criterion was that the value should be lower than the mean minus two times the SD of the control wells. These threshold values for the respective stages were: p.lymf (>0.75), p.bcells (>0.43), p.cd27 (≤0.73), p.cd38 (≤0.71). For the second criterion, a Wilcoxon rank‐sum test was performed for the three replicate values of a compound against the population of positive controls of all three replicates. The resulting *p*‐value was corrected for multiple testing using the Benjamin‐Hochberg method. As a criterion, the corrected *p*‐value was set at a threshold of values lower or equal to 0.1. For a decrease in immunoglobulin production, the criterion was a value <1 and a padj ≤0.1 (*p*‐value adjusted for multiple testing) for all immunoglobulins: IgM, IgG and IgA. All calculations were done in R (version 3.1).

## Authorship contributions

PT and D.J.a.d.K designed the research, performed experiments, analyzed data and wrote the manuscript. M.H.J., B.M. performed experiments. C.L. analyzed data. R.B. designed the research. E.M.M.v.L, T.W.K. designed the research and wrote the manuscript

## Conflict of interest

The authors declare no commercial or financial conflict of interest.

**Abbreviations**
APDS: activated PI3Kδ syndrome
MMF: mycophenolate mofetil
mTOR: mammalian target of rapamycin
PI3K: phosphoinositide 3‐kinase
SLE: systemic lupus erythematosus

## Supporting information

Supporting Information
