# Supplementary material for: High‐throughput compound screen reveals mTOR inhibitors as potential therapeutics to reduce (auto)antibody production by human plasma cells
Source: Eur J Immunol. 2019 Nov 14;50(1):73–85. doi: 10.1002/eji.201948241 (PMC6972998; doi:10.1002/eji.201948241)
Supplement: Supplementary file 1 — Supporting Information [file EJI-50-73-s001.pdf]

European Journal of Immunology

Supporting Information
for
DOI 10.1002/eji.201948241

Paul Tuijnenburg, Daan J. aan de Kerk, Machiel H. Jansen, Ben Morris, Cor Lieftink, Roderick L. Beijersbergen, Ester M.M. van Leeuwen and Taco W. Kuijpers

High-throughput compound screen reveals mTOR inhibitors as potential therapeutics to reduce (auto)antibody production by human plasma cells

© 2019 The Authors. *European Journal of Immunology* published by WILEY-VCH Verlag GmbH & Co. KGaA, Weinheim

Supplemental Figures

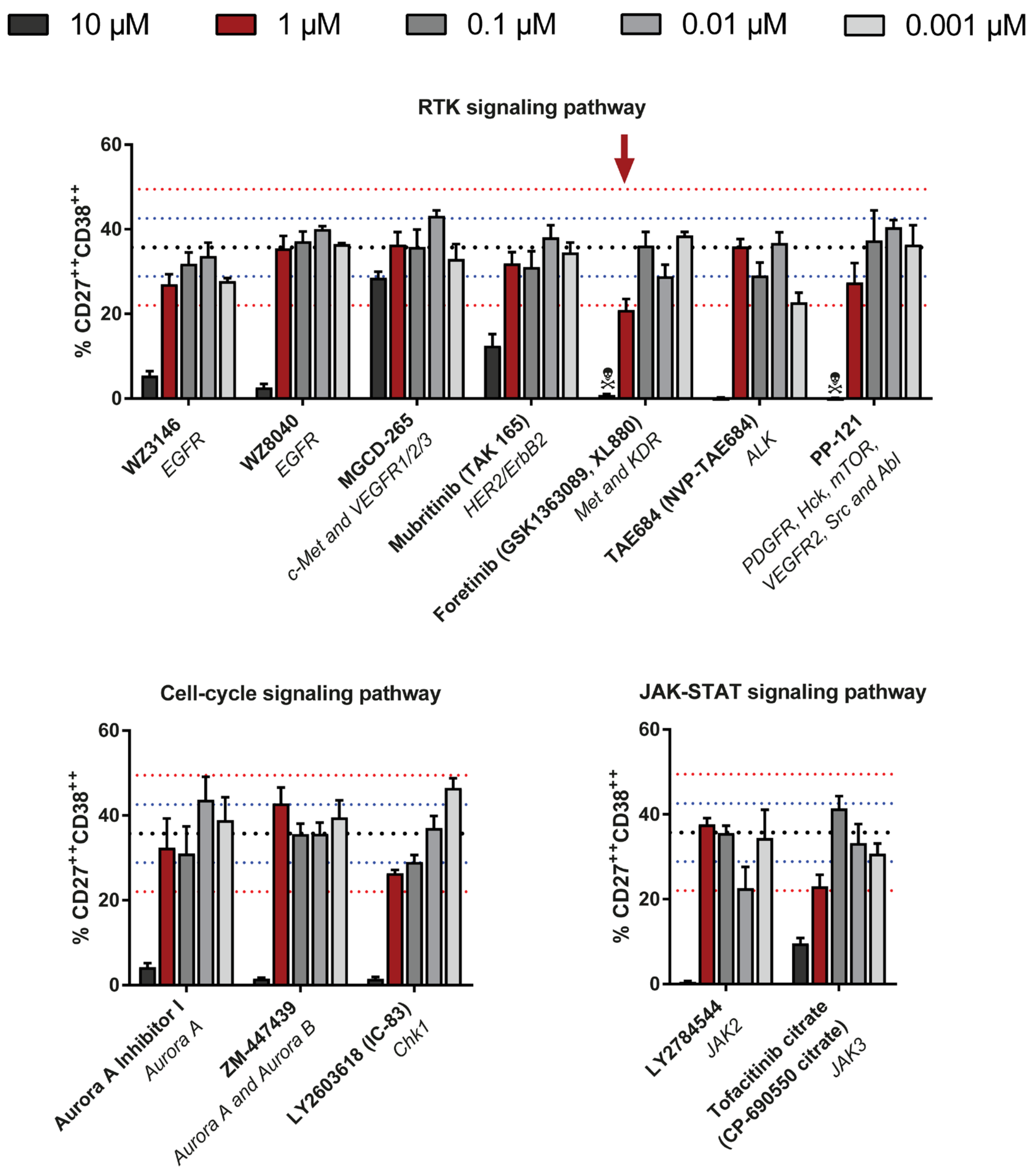

Supplemental Figure 1. Validation of plasmablast-inhibiting compounds, additional pathways. Shown are the pathways not displayed in **Figure 2**. Plasmablast-inhibiting compounds of the initial screening were validated in multiple concentrations around the initial dose of 1 µM. Again, PBMCs were stimulated with CpG/IL-2 for 6 days. Plasmablasts were gated as $CD19^{+}CD20^{dim/+}CD27^{++}CD38^{++}$. *Black dotted line*: mean percentage of $CD27^{++}CD38^{++}$ B cells after 6 days of CpG/IL-2 stimulation without compound (n=72). *Blue dotted line*: ± 1 SD. *Red dotted line*: ± 2 SD. *Red arrow:* Percentage of $CD27^{++}CD38^{++}$ B cells below -2 SD of CpG/IL-2 stimulated cells without compound at the concentration used in the initial screen (1µM). *Toxicity symbol*: Percentage of lymphocytes below -2 SD of CpG/IL-2 stimulated cells without compound.

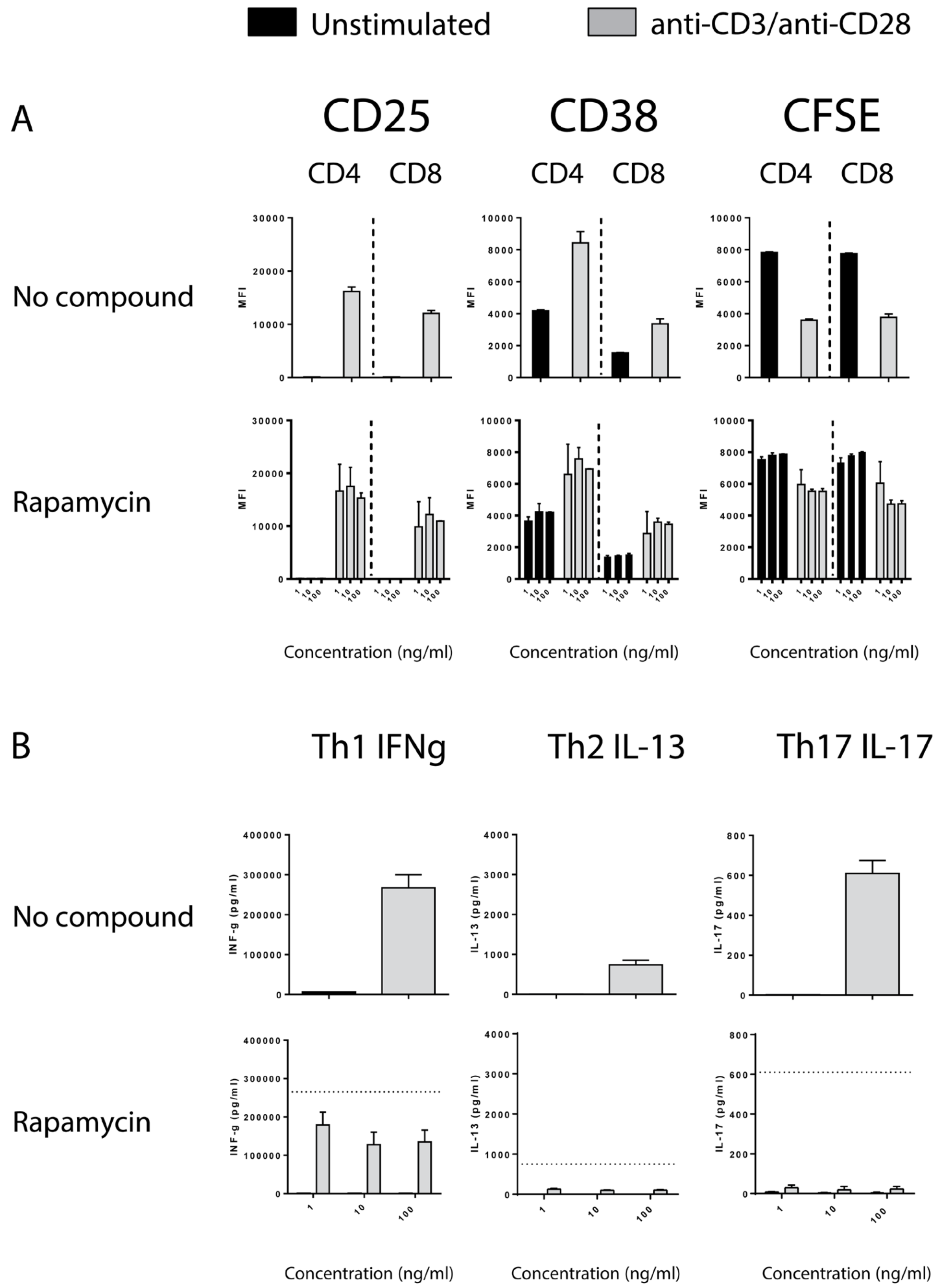

Supplemental Figure 2. **T cell effects of rapamycin.** T cell activation upon stimulation with the combination of anti-CD3/anti-CD28 with and without the addition of rapamycin **(A)** Proliferation by CFSE dilution and upregulation of activation markers CD25 and CD38, measured by MFI shown separately for $CD4^+$ and $CD8^+$ T cells. **(B)** ELISA of cytokine production (in pg/ml) in the supernatant of grouped control experiments (*n*=5) and rapamycin (*n*=2). IFN-γ, IL-13 and IL-17, specific for Th1, Th2 and Th17 T cell subsets, respectively, are shown.

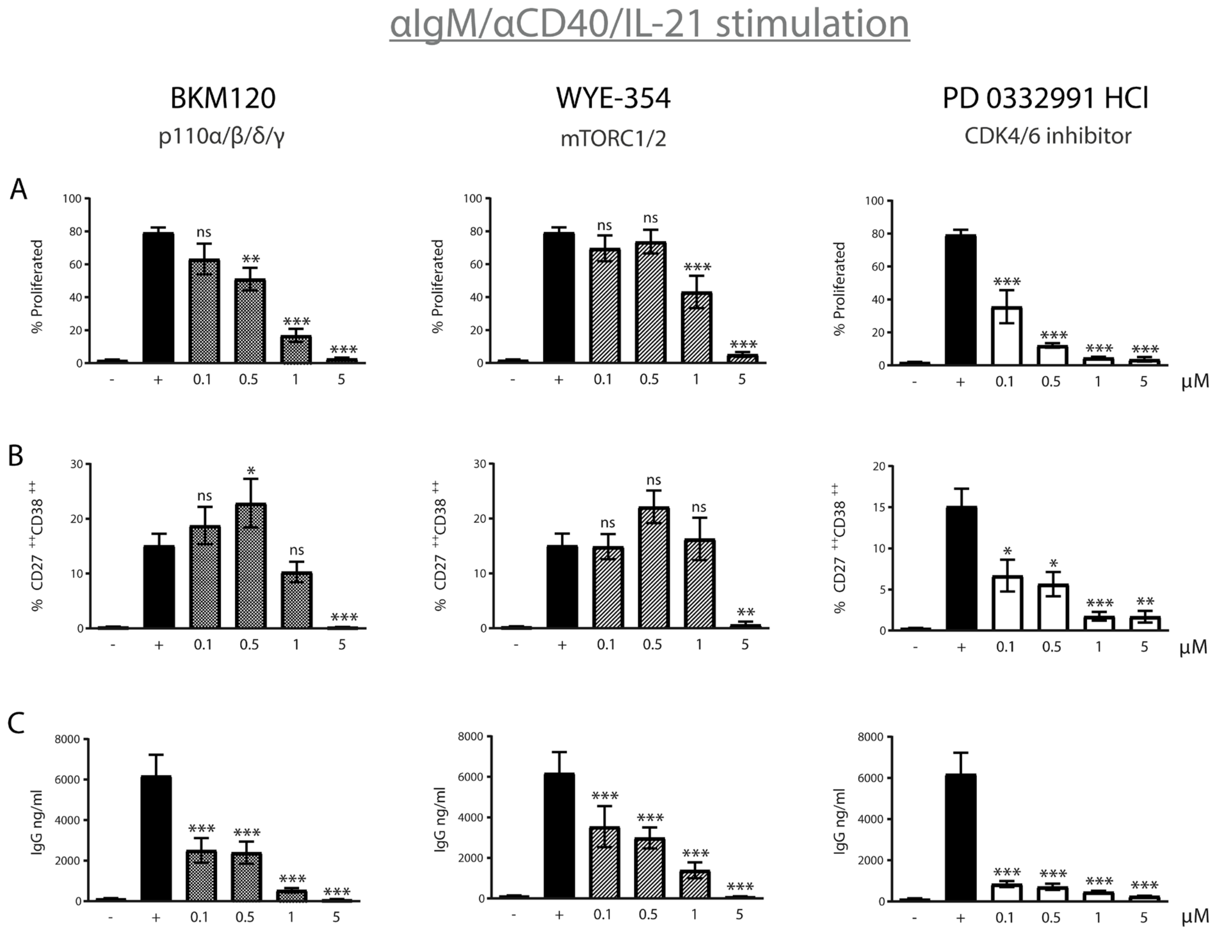

Supplemental Figure 3. BKM120 and WYE-354 similarly reduce immunoglobulin production without reducing plasmablast formation upon T cell dependent activation signals. Proliferation, plasmablast differentiation and immunoglobulin production for BKM120 (left), WYE-354 (middle) and PD 0332991 (right) after 6 days of anti-IgM/anti-CD40/IL-21 stimulation. Summary graphs of multiple flow cytometry experiments for **(A)** percentage proliferated B cells and **(B)** percentage $CD27^{++}CD38^{++}$ plasmablasts. Immunoglobulin production measured by ELISA in 6-day culture supernatant for **(C)** IgG. Symbols: − unstimulated, + stimulated with anti-IgM/anti-CD40/IL-21. Mean ± SEM (for concentrations 0.1 - 1 µM *n*=6 to 10, concentration 5 µM *n*=4). P-values were determined by one-way ANOVA, *ns* not significant, *P≤0.05, **P≤0.01, ***P≤0.001.

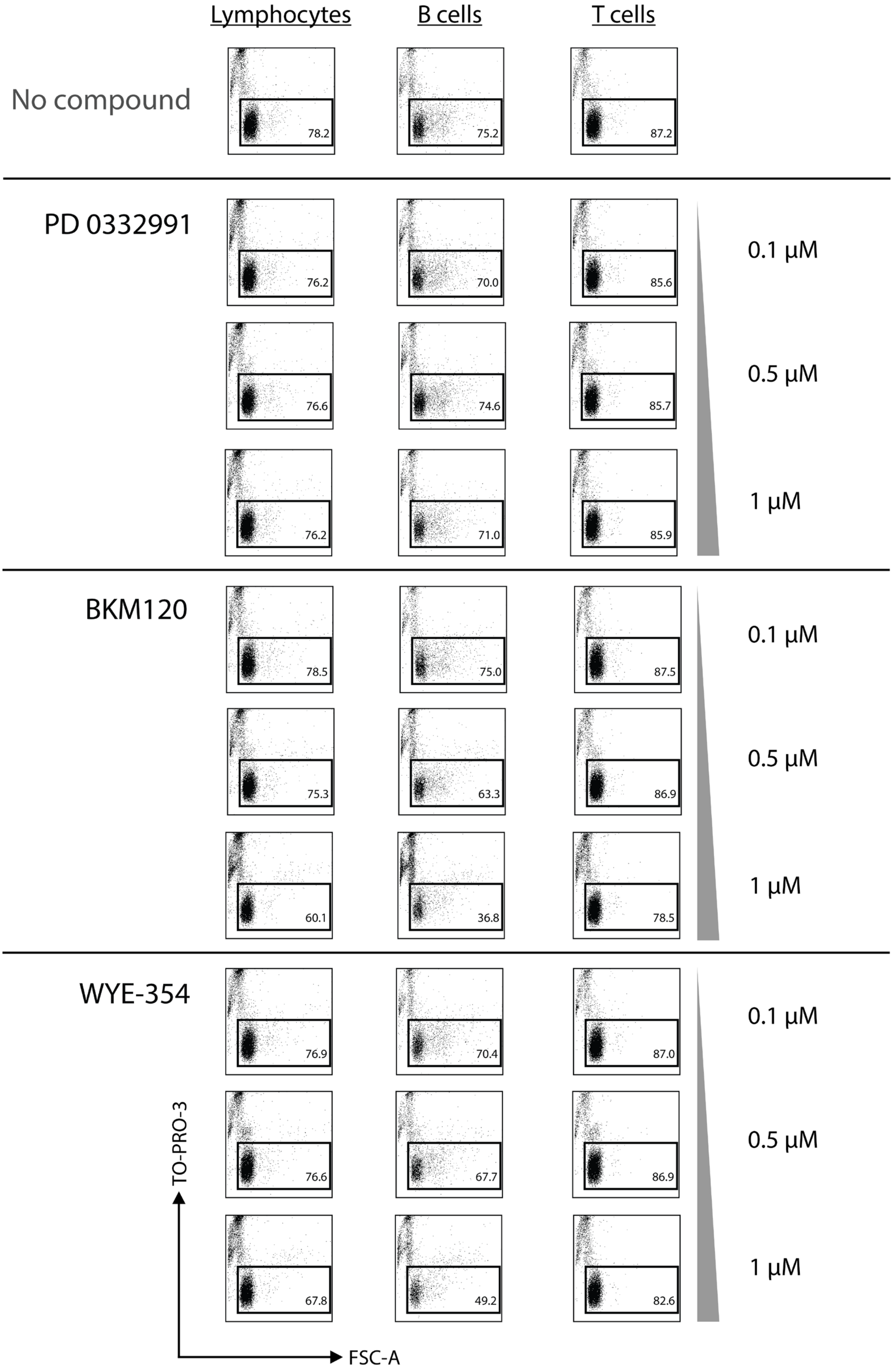

Supplemental Figure 4. Cytotoxicity of compounds PD 0332991, BKM120 and WYE-354. PBMCs were kept in culture for 6 days and different concentrations of compound were added, 0.1 – 1 µM. TO-PRO-3 was measured as a read-out for cytotoxicity, gated on total lymphocytes, CD19^{+} B cells or CD3^{+} T cells.

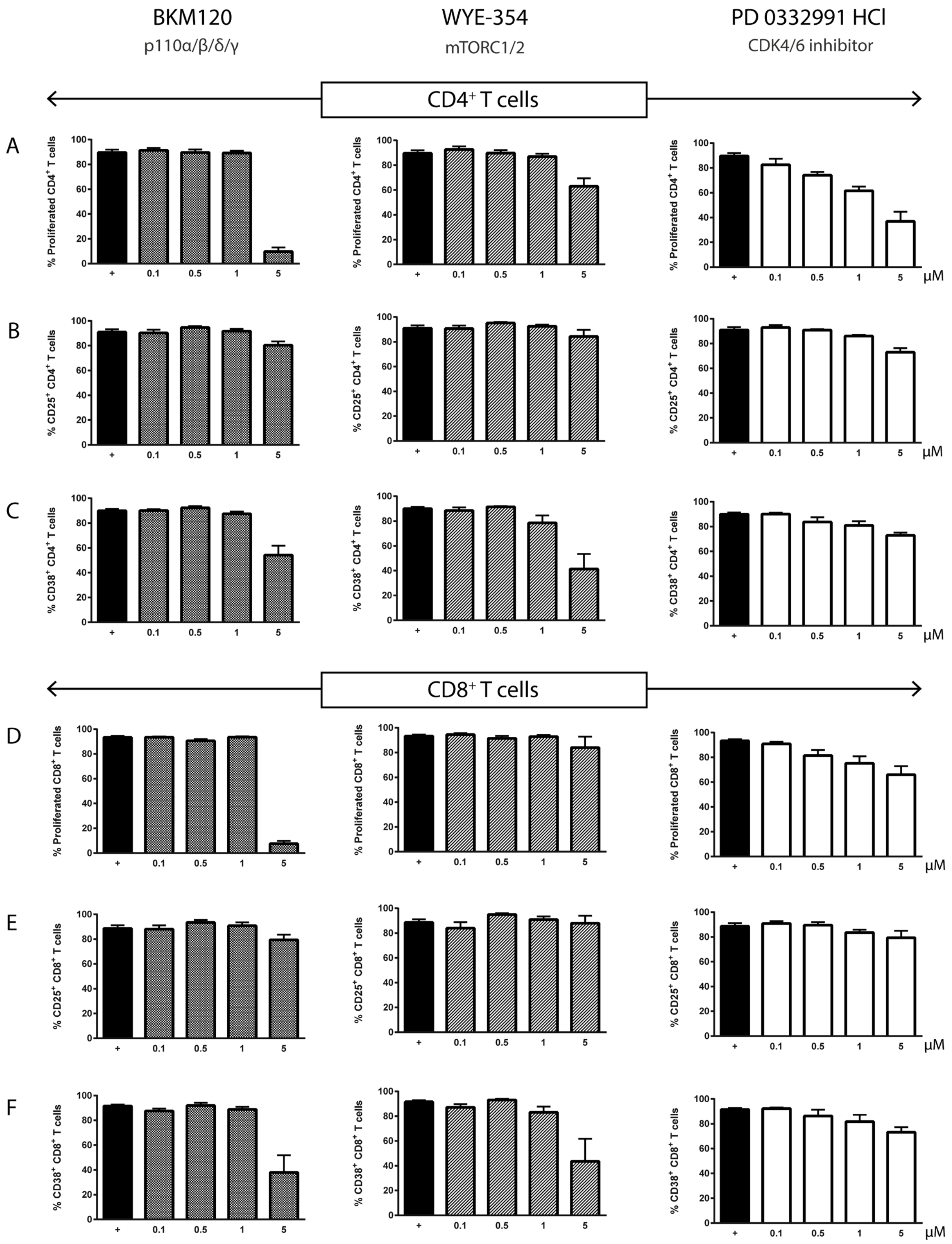

Supplemental Figure 5. Effects on T cell proliferation and activation of PD 0332991, BKM120 and WYE-354 . Effect of compounds on T cell proliferation (CFSE) and activation (CD25 and CD38) after 6 days of anti-CD3/anti-CD28 stimulated PBMCs with the addition of BKM120 (left), WYE-354 (middle) and PD 0332991 (right), shown separately for $CD4^+$ and $CD8^+$ T cells. Different concentrations of compound were added, 0.1 – 5 µM. Symbols: + stimulated with anti-CD3/anti-CD28. Mean ± SEM (for all concentrations *n*=4 to 8).

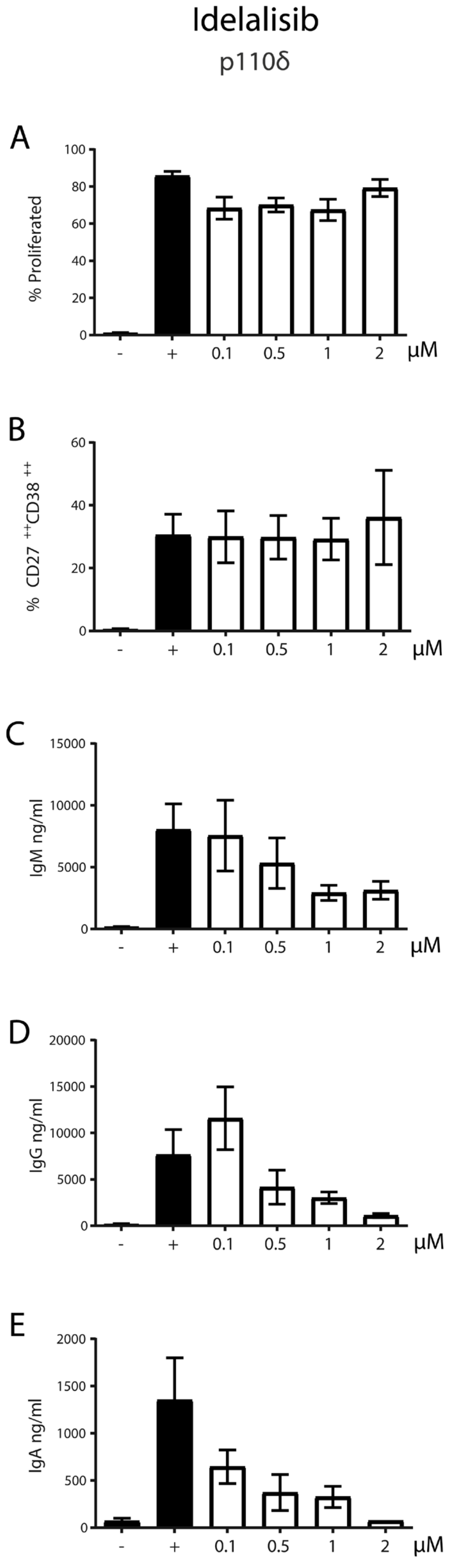

Supplemental Figure 6. Idelalisib shows similar characteristics at lower concentrations as BKM120 and WYE-354. Proliferation, plasmablast differentiation and immunoglobulin production for Idelalisib after 6 days of CpG/IL-2 stimulation. Summary graphs of multiple flow cytometry experiments for **(A)** percentage proliferated B cells and **(B)** percentage $CD27^{++}CD38^{++}$ plasmablasts. Immunoglobulin production measured by ELISA in 6-day culture supernatant for **(C)** IgM, **(D)** IgG and **(E)** IgA. Symbols: − unstimulated, + stimulated with CpG/IL-2. Mean ± SEM (for all concentrations n=3 to 7).

Supplemental Table

Compound Names	Inhibits[1]	Pathway
A66	p110α	PI3K-AKT-mTOR-signaling
A-674563	AKT1	PI3K-AKT-mTOR-signaling
A-769662	reversible AMPK activator	PI3K-AKT-mTOR-signaling
AEE788 (NVP-AEE788)	EGFR and HER2/ErbB2	JAK-STAT-signaling
Afatinib (BIBW2992)	EGFR/HER2	JAK-STAT-signaling
AG-490	EGFT	JAK-STAT-signaling
**AMG 900**	**pan-Aurora kinase**	**Cell-cycle-signaling**
Amuvatinib (MP-470)	C-Kit, PDGFRα and Flt3	Protein Tyrosine Kinase signaling
Apatinib (YN968D1)	VEGFR2	Angiogenesis-signaling
AS-252424	PI3Kγ	PI3K-AKT-mTOR-signaling
AS-604850	PI3Kγ	PI3K-AKT-mTOR-signaling
AS-605240	PI3Kγ	PI3K-AKT-mTOR-signaling
AS703026	MEK1/2	MAPK-signaling
AT7519	multi-CDK	Cell-cycle-signaling
**AT7867**	**AKT1/2/3 and p70S6K/PKA**	**PI3K-AKT-mTOR-signaling**
**AT9283**	**JAK2/3**	**JAK-STAT-signaling**
**Aurora A Inhibitor I**	**Aurora A**	**Cell-cycle-signaling**
Axitinib	VEGFR1, VEGFR2,VEGFR3	Angiogenesis-signaling
**AZ 960**	**JAK2**	**JAK-STAT-signaling**
AZD1480	JAK2	JAK-STAT-signaling
AZD5438	CDK1/2/9	Cell-cycle-signaling
AZD6244 (Selumetinib)	MEK1	MAPK-signaling
AZD7762	Chk1	Cell-cycle-signaling
**AZD8055**	**mTOR**	**PI3K-AKT-mTOR-signaling**
**AZD8330**	**MEK1/2**	**MAPK-signaling**
AZD8931	EGFR, ErbB2 and ErbB3	Angiogenesis-signaling
**Barasertib (AZD1152-HQPA)**	**Aurora B**	**Cell-cycle-signaling**
**BEZ235 (NVP-BEZ235)**	**PI3K and mTOR**	**PI3K-AKT-mTOR-signaling**
BI 2536	Plk1	Cell-cycle-signaling
BI6727 (Volasertib)	Plk1	Cell-cycle-signaling
BIBF1120 (Vargatef)	VEGFR1/2/3, FGFR1/2/3 and PDGFRα/β	Angiogenesis-signaling
**BIRB 796 (Doramapimod)**	**pan-p38 MAPK**	**MAPK-signaling**
BIX 02188	MEK5	MAPK-signaling
BIX 02189	MEK5	MAPK-signaling
**BKM120 (NVP-BKM120, Buparlisib)**	**p110α/β/δ/γ**	**PI3K-AKT-mTOR-signaling**
BMS 777607	c-Met, Axl, Ron and Tyro3	Protein Tyrosine Kinase signaling
BMS 794833	Met/VEGFR2	Protein Tyrosine Kinase signaling
BMS-599626 (AC480)	HER1 and HER2	Protein Tyrosine Kinase signaling
**Bosutinib (SKI-606)**	**Src/Abl**	**Angiogenesis-signaling**
BS-181 HCl	CDK7	Cell-cycle-signaling
**CAL-101 (GS-1101)**	**p110δ**	**PI3K-AKT-mTOR-signaling**
CCT128930	AKT2	PI3K-AKT-mTOR-signaling
CCT129202	Aurora A, Aurora B and Aurora C	Cell-cycle-signaling
Cediranib (AZD2171)	VEGFR(KDR)	Angiogenesis-signaling
CHIR-99021 (CT99021)	GSK-3α/β	PI3K-AKT-mTOR-signaling
CI-1033 (Canertinib)	pan-ErbB	Protein Tyrosine Kinase signaling
CI-1040 (PD184352)	MEK1/2	MAPK-signaling

CP-724714	HER2/ErbB2	Angiogenesis-signaling
Crenolanib (CP-868596)	PDGFRα/β	Angiogenesis-signaling
Crizotinib (PF-02341066)	c-Met and ALK	Protein Tyrosine Kinase signaling
CX-4945	CK2 (casein kinase 2)	Cell metabolism
**CYC116**	**Aurora A/B**	**Cell-cycle-signaling**
**Cyt387**	**JAK1/JAK2**	**JAK-STAT-signaling**
**Danusertib (PHA-739358)**	**Aurora A/B/C**	**Cell-cycle-signaling**
Dasatinib (BMS-354825)	Abl, Src and c-Kit	Angiogenesis-signaling
DCC-2036 (Rebastinib)	Abl1	Angiogenesis-signaling
**Deforolimus (Ridaforolimus)**	**mTOR**	**PI3K-AKT-mTOR-signaling**
**ENMD-2076**	**Aurora A and Flt3**	**Angiogenesis-signaling**
Enzastaurin (LY317615)	PKCβ	TGF-beta/SMAD
Erlotinib HCl	EGFR	Angiogenesis-signaling
Everolimus (RAD001)	mTOR FKBP12	PI3K-AKT-mTOR-signaling
Flavopiridol (Alvocidib)	CDK1, CDK2, CDK4 and CDK6	Cell-cycle-signaling
Flavopiridol hydrochloride	CDK1, CDK2, CDK4 and CDK6	Cell-cycle-signaling
**Foretinib (GSK1363089, XL880)**	**Met and KDR**	**Protein Tyrosine Kinase signaling**
GDC-0879	B-Raf	MAPK-signaling
**GDC-0941**	**PI3Kα/δ**	**PI3K-AKT-mTOR-signaling**
GDC-0980 (RG7422)	PI3Kα/β/δ/γ	PI3K-AKT-mTOR-signaling
Gefitinib (Iressa)	EGFR	Angiogenesis-signaling
GSK1059615	PI3Kα/β/δ/γ and mTOR	PI3K-AKT-mTOR-signaling
**GSK1120212 (Trametinib)**	**MEK1/2**	**MAPK-signaling**
GSK1838705A	IGF-1R	Angiogenesis-signaling
GSK2126458	p110α/β/δ/γ	PI3K-AKT-mTOR-signaling
**GSK461364**	**Plk1**	**Cell-cycle-signaling**
**Hesperadin**	**Aurora B**	**Cell-cycle-signaling**
**HMN-214**	**Plk1**	**Cell-cycle-signaling**
IC-87114	PI3Kδ	PI3K-AKT-mTOR-signaling
Imatinib (Gleevec)	v-Abl, c-Kit and PDGFR	Angiogenesis-signaling
Imatinib Mesylate	v-Abl, c-Kit and PDGFR	Angiogenesis-signaling
Indirubin	GSK-3β	PI3K-AKT-mTOR-signaling
JNJ-38877605	c-Met	Protein Tyrosine Kinase signaling
JNJ-7706621	pan-CDK	Cell-cycle-signaling
Ki8751	VEGFR2	Angiogenesis-signaling
KRN 633	VEGFR1/2/3	Angiogenesis-signaling
**Ku-0063794**	**mTORC1 and mTORC2**	**PI3K-AKT-mTOR-signaling**
KU-55933	ATM	PI3K-AKT-mTOR-signaling
KU-60019	ATM	PI3K-AKT-mTOR-signaling
**KW 2449**	**Flt3**	**Angiogenesis-signaling**
**KX2-391**	**Src**	**Angiogenesis-signaling**
Lapatinib Ditosylate (Tykerb)	EGFR and ErbB2	Angiogenesis-signaling
LDN193189	BMP	TGF-beta/SMAD
Linifanib (ABT-869)	VEGFR/PDGFR	Angiogenesis-signaling
**LY2228820**	**p38 MAPK**	**MAPK-signaling**
**LY2603618 (IC-83)**	**Chk1**	**Cell-cycle-signaling**
**LY2784544**	**JAK2**	**JAK-STAT-signaling**
LY294002	PI3Kα/δ/β	PI3K-AKT-mTOR-signaling
Masitinib (AB1010)	Kit and PDGFRα/β	Protein Tyrosine Kinase signaling

**MGCD-265**	**c-Met and VEGFR1/2/3**	**Protein Tyrosine Kinase signaling**
MK-2206 dihydrochloride	AKT1/2/3	PI3K-AKT-mTOR-signaling
**MLN8237 (Alisertib)**	**Aurora A**	**Cell-cycle-signaling**
Motesanib Diphosphate	VEGFR1/2/3	Angiogenesis-signaling
**Mubritinib (TAK 165)**	**HER2/ErbB2**	**Angiogenesis-signaling**
Neratinib (HKI-272)	HER2 and EGFR	Angiogenesis-signaling
Nilotinib (AMN-107)	Bcr-Abl	Angiogenesis-signaling
NU7441(KU-57788)	DNA-PK	PI3K-AKT-mTOR-signaling
NVP-ADW742	IGF-1R	Protein Tyrosine Kinase signaling
NVP-BHG712	EphB4	Protein Tyrosine Kinase signaling
NVP-BSK805	JAK2	JAK-STAT-signaling
**ON-01910**	**PLK1**	**Cell-cycle-signaling**
OSI-027	mTORC1 and mTORC2	PI3K-AKT-mTOR-signaling
OSI-420 (Desmethyl Erlotinib)	EGFR	Angiogenesis-signaling
OSI-930	Kit, KDR and CSF-1R	Protein Tyrosine Kinase signaling
Pazopanib HCl	VEGFR1, VEGFR2, VEGFR3	Angiogenesis-signaling
**PCI-32765 (Ibrutinib)**	**BTK**	**Angiogenesis-signaling**
**PD 0332991 HCl**	**CDK4/6**	**Cell-cycle-signaling**
PD0325901	MEK	MAPK-signaling
PD153035 HCl	EGFR	Angiogenesis-signaling
PD318088	MEK1/2	MAPK-signaling
PD98059	MEK	MAPK-signaling
Pelitinib (EKB-569)	EGFR	Angiogenesis-signaling
PF-00562271	FAK	Angiogenesis-signaling
PF-04217903	c-Met	Protein Tyrosine Kinase signaling
PF-04691502	PI3K(α/β/δ/γ)/mTOR	PI3K-AKT-mTOR-signaling
**PF-05212384 (PKI-587)**	**PI3Kα, PI3Kγ and mTOR**	**PI3K-AKT-mTOR-signaling**
**PH-797804**	**p38α MAPK**	**MAPK-signaling**
**PHA-680632**	**Aurora A, Aurora B and Aurora C**	**Cell-cycle-signaling**
PHA-793887	CDK2, CDK5 and CDK7	Cell-cycle-signaling
Phenformin hydrochloride	AMPK	PI3K-AKT-mTOR-signaling
PI-103	p110α/β/δ/γ	PI3K-AKT-mTOR-signaling
PIK-293	PI3Kδ	PI3K-AKT-mTOR-signaling
PIK-294	p110δ	PI3K-AKT-mTOR-signaling
PIK-75	p110α	PI3K-AKT-mTOR-signaling
PIK-90	PI3Kα/γ/δ	PI3K-AKT-mTOR-signaling
**PIK-93**	**PI4K (PI4KIIIβ)**	**PI3K-AKT-mTOR-signaling**
PLX-4720	B-Raf	MAPK-signaling
**Ponatinib (AP24534)**	**Abl, PDGFRα, VEGFR2, FGFR1 and Src**	**Angiogenesis-signaling**
**PP-121**	**PDGFR, Hck, mTOR, VEGFR2, Src and Abl**	**Protein Tyrosine Kinase signaling**
**PP242**	**mTOR**	**PI3K-AKT-mTOR-signaling**
Quercetin (Sophoretin)	PI3K and SIRT1	PI3K-AKT-mTOR-signaling
**Quizartinib (AC220)**	**FLT3**	**Angiogenesis-signaling**
R406	Syk	Angiogenesis-signaling
R406(free base)	Syk	Angiogenesis-signaling
R935788 (Fostamatinib disodium, R788)	Syk	Angiogenesis-signaling
**RAF265 (CHIR-265)**	**C-Raf/B-Raf/B-Raf V600E**	**MAPK-signaling**
Raf265 derivative	C-Raf/B-Raf/B-Raf V600E	MAPK-signaling

**Rapamycin (Sirolimus)**	**mTOR**	**PI3K-AKT-mTOR-signaling**
Regorafenib (BAY 73-4506)	VEGFR1/2/3 PDGFRβ, Kit, RET and Raf-1	Protein Tyrosine Kinase signaling
Roscovitine (Seliciclib, CYC202)	Cdc2, CDK2 and CDK5	Cell-cycle-signaling
Saracatinib (AZD0530)	Src	Angiogenesis-signaling
**SB 202190**	**MAPK**	**MAPK-signaling**
SB 203580	MAPK	MAPK-signaling
SB 216763	GSK-3	PI3K-AKT-mTOR-signaling
SB 431542	ALK5	TGF-beta/SMAD
SB 525334	ALK5	TGF-beta/SMAD
SGX-523	Met	Protein Tyrosine Kinase signaling
SNS-032 (BMS-387032)	CDK2	Cell-cycle-signaling
**SNS-314**	**Aurora A, Aurora B and Aurora C**	**Cell-cycle-signaling**
Sorafenib (Nexavar)	Raf-1, B-Raf and VEGFR-2	MAPK-signaling
SP600125	JNK1, JNK2 and JNK3	MAPK-signaling
SU11274	Met	Protein Tyrosine Kinase signaling
Sunitinib Malate (Sutent)	VEGFR2 (Flk-1) and PDGFRβ	Protein Tyrosine Kinase signaling
**TAE684 (NVP-TAE684)**	**Alk**	**Angiogenesis-signaling**
TAK-733	MEK1	MAPK-signaling
**TAK-901**	**Aurora A/B**	**Cell-cycle-signaling**
Tandutinib (MLN518)	FLT3	Angiogenesis-signaling
Telatinib (BAY 57-9352)	VEGFR2/3, c-Kit and PDGFRα	Protein Tyrosine Kinase signaling
**Temsirolimus (Torisel)**	**mTOR**	**PI3K-AKT-mTOR-signaling**
TG100-115	PI3Kγ/δ	PI3K-AKT-mTOR-signaling
TG101209	JAK2	JAK-STAT-signaling
TGX-221	p110β	PI3K-AKT-mTOR-signaling
Tivozanib (AV-951)	VEGFR1/2/3	Protein Tyrosine Kinase signaling
**Tofacitinib citrate (CP-690550 citrate)**	**JAK3**	**JAK-STAT-signaling**
TSU-68	PDGFR	Protein Tyrosine Kinase signaling
U0126-EtOH (UO126 EtOH)	MEK1/2	MAPK-signaling
Vandetanib (Zactima)	VEGFR2	Protein Tyrosine Kinase signaling
Vatalanib dihydrochloride (PTK787)	VEGFR2/KDR	Protein Tyrosine Kinase signaling
**VX-680 (MK-0457, Tozasertib)**	**Aurora A**	**Cell-cycle-signaling**
**VX-702**	**p38α MAPK**	**MAPK-signaling**
**VX-745**	**p38α MAPK**	**MAPK-signaling**
WAY-600	mTOR	PI3K-AKT-mTOR-signaling
**WP1130**	**deubiquitinase**	**Angiogenesis-signaling**
**WYE-125132**	**mTOR**	**PI3K-AKT-mTOR-signaling**
**WYE-354**	**mTOR**	**PI3K-AKT-mTOR-signaling**
WYE-687	mTOR	PI3K-AKT-mTOR-signaling
**WZ3146**	**EGFR**	**Protein Tyrosine Kinase signaling**
WZ4002	EGFR	Protein Tyrosine Kinase signaling
**WZ8040**	**EGFRT790M**	**Protein Tyrosine Kinase signaling**
XL147	PI3Kα/δ/γ	PI3K-AKT-mTOR-signaling
XL-184 free base (Cabozantinib)	VEGFR2	Protein Tyrosine Kinase signaling
XL765	p110γ	PI3K-AKT-mTOR-signaling
ZM 336372	c-Raf	MAPK-signaling
**ZM-447439**	**Aurora A and Aurora B**	**Cell-cycle-signaling**
**ZSTK474**	**PI3K**	**PI3K-AKT-mTOR-signaling**

[1] Kinase Inhibitor Library (Catalog no. L1200) from Selleck Chemicals (Houston, Texas, http://selleckchem.com)
